# The Impact of Cytokines on Coagulation Profile in COVID-19 Patients: Controlled for Socio-Demographic, Clinical, and Laboratory Parameters

**DOI:** 10.3390/biomedicines12061281

**Published:** 2024-06-10

**Authors:** Milica Milentijević, Nataša Katanić, Bojan Joksimović, Aleksandar Pavlović, Jelena Filimonović, Milena Anđelković, Ksenija Bojović, Zlatan Elek, Siniša Ristić, Miloš Vasiljević, Jasmina Stevanović, Danica Radomirović, Nikolina Elez-Burnjaković, Nenad Lalović, Milan Kulić, Jovan Kulić, Marija Milić

**Affiliations:** 1 Department of Infective Diseases, Faculty of Medicine, University of Pristina Temporarily Settled in Kosovska Mitrovica, 38220 Kosovska Mitrovica, Serbia; milica_milosavljevic@hotmail.com (M.M.); katanicn@gmail.com (N.K.); 2 Clinical Hospital Center Kosovska Mitrovica, 38220 Kosovska Mitrovica, Serbia; millena.andjelkovic@gmail.com (M.A.); drzelek@gmail.com (Z.E.); danicarkm@gmail.com (D.R.); 3 Faculty of Medicine Foča, University of East Sarajevo, 73300 Foča, Republic of Srpska, Bosnia and Herzegovina; ksenijabojovi@gmail.com (K.B.); risticsinisa@yahoo.com (S.R.); vasiljevicmilos85@gmail.com (M.V.); nikolinaa85@hotmail.com (N.E.-B.); nenad.lalovic@gmail.com (N.L.); kulicmilan62@gmail.com (M.K.); kulicjovan@yahoo.com (J.K.); 4 Department of Surgery, Faculty of Medicine, University of Pristina Temporarily Settled in Kosovska Mitrovica, 38220 Kosovska Mitrovica, Serbia; sasaaleksandarpavlovic@gmail.com; 5 Department of Epidemiology, Faculty of Medicine, University of Pristina Temporarily Settled in Kosovska Mitrovica, 38220 Kosovska Mitrovica, Serbia; jfilimonovic75@gmail.com (J.F.); jaska.stevanovic@gmail.com (J.S.)

**Keywords:** COVID-19, cytokines, coagulation parameters, D-dimer, fibrinogen, aPTT

## Abstract

**Background:** Severe coagulation abnormalities are common in patients with COVID-19 infection. We aimed to investigate the relationship between pro-inflammatory cytokines and coagulation parameters concerning socio-demographic, clinical, and laboratory characteristics. **Methods:** Our study included patients hospitalized during the second wave of COVID-19 in the Republic of Serbia. We collected socio-demographic, clinical, and blood-sample data for all patients. Cytokine levels were measured using flow cytometry. **Results:** We analyzed data from 113 COVID-19 patients with an average age of 58.15 years, of whom 79 (69.9%) were male. Longer duration of COVID-19 symptoms before hospitalization (B = 69.672; *p* = 0.002) and use of meropenem (B = 1237.220; *p* = 0.014) were predictive of higher D-dimer values. Among cytokines, higher IL-5 values significantly predicted higher INR values (B = 0.152; *p* = 0.040) and longer prothrombin times (B = 0.412; *p* = 0.043), and higher IL-6 (B = 0.137; *p* = 0.003) predicted longer prothrombin times. Lower IL-17F concentrations at admission (B = 0.024; *p* = 0.050) were predictive of higher INR values, and lower IFN-γ values (B = −0.306; *p* = 0.017) were predictive of higher aPTT values. **Conclusions:** Our findings indicate a significant correlation between pro-inflammatory cytokines and coagulation-related parameters. Factors such as the patient’s level of education, gender, oxygen-therapy use, symptom duration before hospitalization, meropenem use, and serum concentrations of IL-5, IL-6, IL-17F, and IFN-γ were associated with worse coagulation-related parameters.

## 1. Introduction

COVID-19 infection has resulted in over seven million deaths worldwide, primarily due to damage to multiple organs [1,2]. The SARS-CoV-2 virus enters host cells through a receptor called angiotensin-converting enzyme 2 (ACE2), which is present in various human organs, making them susceptible to the virus [1,2,3,4,5]. Initially, the virus directly damages infected cells; this is followed by a strong inflammatory response in later stages, leading to excessive production of pro-inflammatory cytokines and thus worsening disease symptoms [1].

While most COVID-19 patients initially show respiratory symptoms, some develop severe systemic illness characterized by persistent high fever, pneumonia, acute respiratory distress syndrome (ARDS), and multiorgan dysfunction, with ARDS being a leading cause of death [6]. Some patients experience a cytokine-storm syndrome wherein the immune response produces excessive pro-inflammatory cytokines that are known to activate blood-clotting pathways and cause organ damage [7,8]. The pathophysiology of ARDS in COVID-19 shares similarities with that of pneumonia caused by other viruses and bacteria [9].

Pro-inflammatory cytokines such as interleukin (IL)-6, IL-2, IL-7, IL-1β, and tumor necrosis factor α (TNF-α) are produced excessively in COVID-19 patients, leading to a cytokine storm. This storm increases the risk of blood-clotting disorders, multiorgan dysfunction, and death [7,10,11,12]. For instance, IL-6 prompts the expression of tissue factors on certain cells, promoting blood-clot formation [8]. Similarly, TNF-α and IL-1β regulate the activity of the blood-clotting system [8].

Coagulation abnormalities are a significant feature of COVID-19, affecting many patients [3,13]. COVID-19-associated coagulopathy (CAC) is characterized by an increased tendency toward blood clotting and a heightened risk of blood-vessel blockages [3]. CAC arises from various factors, including activation of blood clotting, suppression of natural clot-breakdown mechanisms, increased blood thickness due to low oxygen levels, and blood-vessel constriction [3,14,15]. Hospitalized COVID-19 patients, particularly those in intensive care, are at a higher risk of developing blood clots, commonly leading to deep vein thrombosis and pulmonary embolism [3].

D-dimer, a product of blood-clot breakdown, is a key marker of blood-clotting status in COVID-19 patients [3]. Elevated D-dimer levels indicate a poorer prognosis with a higher likelihood of severe illness and death [3]. Additionally, other coagulation markers such as platelet count, prothrombin time (PT), activated partial thromboplastin time (aPTT), international normalized ratio (INR), and fibrinogen should be regularly monitored in hospitalized COVID-19 patients [3,16]. Despite extensive research on the association between coagulation markers and COVID-19 severity and outcomes, there are some discrepancies in the findings [17]. Furthermore, the relationship between coagulation status and cytokine levels remains inadequately studied [18,19].

Given the excessive production of pro-inflammatory cytokines during the immune response to SARS-CoV-2, which activates blood clotting pathways, it is crucial to identify the specific cytokines involved in the cytokine storm and understand how their secretion relates to coagulation parameters, potentially leading to blood-clotting disorders and blockages in small blood vessels. Therefore, this study aims to investigate the connections between pro-inflammatory cytokines, coagulation parameters, and various socio-demographic, clinical, and laboratory factors.

## 2. Materials and Methods

### 2.1. Study Design and Data Collection

Our study population consisted of COVID-19 patients hospitalized at the Clinical Hospital Center Kosovska Mitrovica, Serbia, during the second wave from July to September 2020. All participants were from Kosovo and Metohija. Prior to participation, all patients received detailed information about the study’s purpose and methodology and provided written informed consent for the study and blood sample collection. The Ethics Committee of Clinical Hospital Center Kosovska Mitrovica and the Faculty of Medicine, University of Pristina, temporarily located in Kosovska Mitrovica, granted permission for this cross-sectional study (ethical permission number 10-1257, issued on 23 July 2020). Based on clinical and diagnostic criteria, we categorized the severity of COVID-19 in our patients as follows:

1. Form 1 (uncomplicated disease): Patients were either asymptomatic or displayed very mild symptoms. These were individuals without any underlying health conditions and with mild infection. Hospitalized patients had oxygen levels (pO_2_) above 94% and no signs of pneumonia on radiographic imaging.
2. Form 2 (moderate disease): Patients exhibited a mild clinical presentation. They did not have any comorbidities and showed mild infection symptoms. Hospitalized patients had oxygen levels (pO_2_) above 94% but displayed signs of pneumonia on radiographic imaging, with or without hypoxia.
3. Form 3 (severe disease): Patients presented with a moderately severe clinical picture. They experienced severe hypoxia requiring oxygen therapy (SpO_2_ < 90%), fever, multiple opacifications on radiographic imaging, and/or specific lung changes detected on CT scans.

For the purpose of our study, we categorized patients into two groups: those with mild/moderate forms of COVID-19 and those with severe forms of the disease.

### 2.2. Data Collection

Patient data, including sociodemographic details, clinical and laboratory test results, were collected upon admission, and a blood sample was taken for measurement of cytokine levels.

We gathered the following socio-demographic and epidemiological information: gender, age, body mass index (BMI), changes in BMI over six months (decrease/no change/increase), relationship status (single/in a relationship/married), level of education (<8 years or elementary school/8–12 years or secondary school/>12 years or higher education), employment status (unemployed/employed/retired), smoking status (non-smoker/ex-smoker/smoker), years of smoking, number of cigarettes per day, alcohol consumption (never/monthly or less often/weekly), bedtime, duration of sleep in hours, changes in sleep duration during COVID-19 disease compared to before (less/more/no change), sleep quality before COVID-19 disease (poor to very poor/average/good to very good), sleep quality during COVID-19 disease (poor to very poor/average/good to very good), use of immune-boosting supplements (no/yes), duration of supplement use before COVID-19 infection, close contact with confirmed COVID-19 cases (no/don’t know/yes), visit to a COVID-19 facility before infection (no/yes), and whether family members had COVID-19 (no/yes).

Additionally, we collected the following clinical data from patients and medical records: number of COVID-19 symptoms per patient (none/one/two/three/four/five or more), duration of symptoms before hospitalization, number of chronic diseases per patient (none/one/two/three or more), oxygen therapy (no/yes), duration of oxygen therapy in days, amount of oxygen administered in liters, high-flow oxygen therapy (no/yes), use of a respirator (no/yes), length of hospitalization in days, severity of clinical presentation (mild to moderate/severe to very severe), COVID-19 outcome (recovered/transferred to another institution/discharge at personal request/death), X-ray findings on admission (no disease/bilateral pneumonia/right-lung pneumonia/left-lung pneumonia), changes in X-ray findings at discharge (no disease or regression/stable findings/progression), affected lung fields (none/upper/lower/middle/upper and middle/lower and middle/all lung fields), lung-auscultation findings (normal/bilateral crackles/unilateral crackles/weakened breath sounds/hardened breath sounds), ECG results (sinus rhythm/arrhythmia/tachycardia/asystole), and therapy administered (symptomatic treatment/anticoagulants/corticosteroids/chloroquine/favipiravir/ceftriaxone/levofloxacin/vancomycin/meropenem/metronidazole/tocilizumab).

### 2.3. Laboratory and Cytokine Analyses

In our analysis, we included a range of laboratory data collected upon admission. We assessed blood parameters including complete blood count with leukocyte formula; glucose, urea, and creatinine levels; cholesterol, albumin, bilirubin, and triglyceride levels; high-density lipoprotein (HDL) and low-density lipoprotein (LDL) levels; aspartate aminotransferase (AST), alanine transaminase (ALT), and gamma-glutamyl transpeptidase (GGT) levels; iron (Fe) levels; sodium (Na), potassium (K), and chlorine (Cl) levels; C-reactive protein (CRP) levels; alkaline reserve levels; prothrombin time (PT); international normalized ratio (INR); activated partial thromboplastin time (aPTT); fibrinogen levels; D-dimer levels; and sedimentation rates.

A blood sample was collected in a 5-mL plastic whole-blood tube that had been spray-coated with 10 mg K_2_ ethylenediaminetetraacetic acid (EDTA; BD Vacutainer, Franklin Lakes, NJ, USA). One ml of the whole blood was used for determining complete blood count, while the rest of this sample was left at room temperature (RT) for 30 min, centrifuged at 3000 rpm at RT for 10 min, divided into 250 μL aliquots of serum in 0.5 mL polypropylene storage tubes (Sarstedt, Nümbrecht, Germany), stored at −80 °C, and later used for previously mentioned analyses and for measuring the levels of various pro-inflammatory and anti-inflammatory serum cytokines. These included interleukin (IL)-2, IL-4, IL-5, IL-6, IL-9, IL-10, IL-13, IL-17A, IL-17F, IL-21, IL-22, interferon-gamma (IFN-γ), and tumor necrosis factor alpha (TNF-α). These cytokine levels were assessed using a flow cytometer (Attune Acoustic Focusing Cytometer, Applied Biosystems, Thermo Fisher, Carlsbad, CA, USA) with fluorescently labeled beads containing anti-cytokine antibodies (Biolegend, San Diego, CA, USA).

### 2.4. Statistical Analysis

We used the SPSS Windows user package, version 19, to analyze the data. First, the data were analyzed using descriptive statistics methods. The mean value and standard deviation were used for continuous variables, while the frequency (percentage) was used for categorical variables. We established *p* < 0.05 as the statistical significance level. The Kruskal–Wallis KW χ^2^ test and the Mann–Whitney U test were used to examine the differences between variables. Spearman’s correlation coefficient was used to test the relationship between the variables. The predictive potential of the variables that showed statistical significance in the univariate analysis on the outcome variables of interest (coagulation parameters) was investigated using linear regression analysis.

## 3. Results

### 3.1. Socio-Demographic Characteristics and Their Correlation with Coagulation Status in Patients with COVID-19 Infection

Our research involved 113 COVID-19 patients with an average age of 58.15 ± 13.50 years (mean value ± standard deviation (SD)), of whom 79 (69.9%) were male. Differences in coagulation status and their correlations with the examined parameters are given in Table 1 and Supplementary Tables S1–S3. Analyzing the association of the investigated factors with D dimer levels, the correlation analysis showed a significant weak positive correlation between age and D dimer levels (r = 0.240; *p* < 0.050). D-dimer levels were significantly (*p* < 0.050) higher in patients with a lower level of education (<8 years) (1498.00 ± 2995.62 ng/mL) compared to patients with an intermediate (8–12 years) (411.00 ± 395.40 ng/mL) o higher level of education (>12 years) (709.57 ± 1245.31 ng/mL). When it comes to smoking status, ex-smokers had significantly (*p* < 0.050) higher concentrations of D-dimer (1108.90 ± 2245.89 ng/mL) compared to smokers (268.17 ± 102.14 ng/mL) and non-smokers (487.00 ± 711 ng/mL), as did patients with chronic disease compared to those without (1000.61 ± 2104.58 ng/mL vs. 517.45 ± 872.23 ng/mL; *p* < 0.050) and those not treated with corticosteroids compared to those treated with corticosteroids (693,288 ± 1819.95 ng/mL vs. 575.67 ± 841.97 ng/mL; *p* < 0.050). Correlation analysis showed a significant weak positive association of D-dimer levels with levels of C-reactive protein (r = 0.219; *p* = 0.042), leukocytes (r = 0.296; *p* = 0.006), AST (r = 0.232; *p* = 0.032) and urea (r = 0.234; *p* = 0.031) and a medium-strong association with erythrocyte sedimentation (r = 0.413; *p* = 0.001), while a significant medium-strong negative association was observed between D-dimer and albumin (r = −0.320 *p*= 0.003), IL-5 (r = −0.277; *p* = 0.010) and a significant medium-strong positive association was observed with IL-6 (r = 0.223; *p* = 0.038).

A significant, medium-strong positive correlation was observed between the length of use of supplements before COVID-19 infection and the platelet count (r = 0.389; *p* < 0.050), and a significant weak negative correlation was observed between the duration of symptoms and the platelet count (r = 0.214; *p* < 0.050). Platelets are significantly positively moderately associated with sedimentation of erythrocytes (r = 0.295; *p* = 0.003), leukocytes (r = 0.433; *p* = 0.001), basophils (r = 0.450; *p* = 0.001) and alkaline phosphatase (*p* = 0.216; 0.001), and with levels of IL-17F (r = 0.189; *p* = 0.047).

The mean aPTT value was significantly higher in patients with arrhythmia compared to patients with sinus rhythm (38.00 ± 9.04 s vs. 28.20 ± 3.65 s; *p* < 0.010) and was also significantly higher in patients who did not use corticosteroids (31.41 ± 5.94 s vs. 26.86 ± 2.94 s; *p* < 0.050) and meropenem (29.89 ± 5.45 s vs. 26.00 ± 1.51 s; *p* < 0.050) compared to those who did not. A significant medium-strong positive correlation of aPTT with lymphocytes (r = 0.493; *p* = 0.008) and monocytes (r = 0.438; *p* = 0.0020) was observed, as was a significant strong negative correlation of aPTT with neutrophils (r = −0.526, *p* = 0.004) and IFN-γ (r = −0.537; *p* = 0.003).

Fibrinogen levels were significantly higher in men than women (6.00 ± 1.49 g/L vs. 4.98 ± 1.58 g/L, *p* < 0.010), patients who had no close contact with a confirmed case of COVID-19 compared to those who had (6.19 ± 1.54 g/L vs. 4.92 ± 1.03 g/L, *p* < 0.010), patients who did not visit facilities with COVID-19 before the onset of infection compared to those who visited (5.89 ± 1.52 g/L vs. 4.82 ± 1.59 g/L, *p* < 0.050), patients in whom the X-ray finding showed involvement of all lung fields compared to those without lung disease (8.11 ± 0.00 g/L vs. 4.49 ± 1.02 g/L, *p* < 0.010, patients who received oxygen therapy compared to those who did not (6.25 ± 1.35 g /L vs. 5.33 ± 1.63 g/L; *p* < 0.050) and patients who were treated with corticosteroids compared to patients who were not (6.15 ± 1.51 g/L vs. 5.03 ± 1, 46 g/L; *p* < 0.010). A significant weak negative correlation was observed between the concentration of fibrinogen and the duration of symptoms before hospitalization (r = −0.269; *p* < 0.050); the concentration of fibrinogen also showed a moderate negative correlation with monocytes (r = −0.301; *p* = 0.013), eosinophils (r = −0.469; *p*= 0.001), albumins (r = −0.293; *p* = 0.015), Na+ (r = −0.307; *p* = 0.010) and Fe (r = −0.457; *p* =0.002) and a strong negative association with lymphocytes (r = −0.593; *p* = 0.001). Fibrinogen was significantly strongly positively associated with C-reactive protein (r = 0.626; *p* = 0.001), erythrocyte sedimentation rate (r = 0.501; *p* = 0.001), neutrophils (r = 0.526; *p* = 0.004), direct bilirubin (0.330; *p* = 0.007), K+ (r = 0.263; *p* = 0.029) and IL-17F (r = 0.258; *p* = 0.033).

Prothrombin time was significantly (*p* < 0.050) longer in single patients (19.69 ± 11.75 s) compared to patients who were in a relationship or married (12.48 ± 0.91 s), in non-smokers (16.81 ± 9.58 s, *p* < 0.050) compared to ex-smokers (12.73 ± 0.55 s) and smokers (11.47 ± 0.75 s), in patients with moderate sleep quality (17.91 ± 11.21 s, *p* < 0.010) compared to patients with poor/very poor sleep (16.15 ± 6.44 s) and good/very good sleep (12.09 ± 0.93 s), in patients who did not use supplements to strengthen immunity (16.65 ± 9.40 s, *p* < 0.050) compared to patients who used immune-boosting supplements (12.23 ± 0.39 s) (Table 1), in arrhythmia patients compared to sinus-rhythm patients (27.50 ± 12.66 vs. 13.73 ± 5.95 s; *p* < 0.001), and in patients treated with vancomycin compared to patients not treated with vancomycin (24.30 ± 17.36 vs. 14.56 ± 0.05 s, *p* < 0.05). Prothrombin time was significantly negatively and moderately strongly associated with levels of basophils (r= −0.451; *p* = 0.021) and cholesterol (r = −0.386; *p* = 0.015) (Supplementary Table S1).

The INR was significantly (*p* < 0.010) higher in highly educated patients (3.67 ± 3.70) compared to patients with medium (1.22 ± 0.36) and low levels of education (1.67 ± 0.48). Non-smokers had significantly (*p* < 0.001) higher average INR values (2.31 ± 2.58) compared to ex-smokers (1.16 ± 0.05) and smokers (1.04 ± 0.07); similar patterns held for patients with moderate sleep quality (2.67 ± 3.15, *p* < 0.050) compared to patients with poor or very poor sleep (1.47 ± 0.59) and patients with good/very good sleep (1.33 ± 0.75); for patients with arrhythmia compared to patients with sinus rhythm (2.52 ± 1.17 g/L vs. 1.88 ± 2.35 g/L; *p* < 0.010); and for patients treated with favipiravir compared to patients who were not treated with favipiravir (2.04 ± 1.94 vs. 1.90 ± 2.50; *p* < 0.050). Analyses showed that INR was significantly negatively and moderately strongly associated with cholesterol levels (r = −0.431; *p* = 0.015) (Table 1).

### 3.2. Differences in the Concentration of Cytokines in Patients with COVID-19 Infection in Relation to the Severity of the Clinical Manifestations

Table 2 shows the average values of cytokine concentration between groups of hospitalized patients with COVID-19 infection, divided according to the severity of the clinical manifestations. Serum concentrations of IL-2 (74.13 ± 14.68 pg/mL vs. 54.35 ± 5.27 pg/mL; *p* = 0.001), IL-6 (157.49 ± 56.33 pg/mL vs. 101.67 ± 19.12 pg/mL; *p* = 0.001), IL-17A (9.48 ± 3.24 pg/mL vs. 7.41 ± 1.60 pg/mL; *p* = 0.001) and TNF-α (50.00 ± 16.00 pg/mL vs. 30.63 ± 19.45 pg/mL; *p* = 0.001) were significantly higher in patients with severe clinical manifestations compared to patients with a mild/moderate clinical manifestations. Differences in the average values of IL-4, IL-5, IL-9, IL-10, IL-13, IL-17F, IL-21, IL-22, and IFN-γ in the serum of patients divided into groups according to the severity of the clinical manifestations were not observed. However, it is noticeable that levels of IL-2, IL-4, IL-5, IL-6, IL-9, IL-10, IL-17F, IL-21, IFN-γ, and TNF-α were higher in COVID-19 patients compared to healthy individuals, where IL-13, IL-17A, and IL-22 were in the normal range (Table 2).

### 3.3. Cytokine Profile as a Predictor of Coagulation Status in a Model Controlled for Socio-Demographic Characteristics in Patients with COVID-19 Infection

Table 3, Table 4 and Table 5 shows the multivariate regression analysis of cytokine profile as a predictor of coagulation status in models controlled for the socio-demographic characteristics (“Socio-Demographic Model”), clinical characteristics (“Clinical Model”), and laboratory parameters (“Laboratory Model”) of hospitalized patients with COVID-19 infection. Variables that showed significance in univariate analysis in relation to coagulation status were analyzed in regression models.

In the Socio-Demographic Model, lower concentrations of IL-17F at admission (B = 0.024; *p* = 0.050) and lower levels of education (B = 2.948; *p* = 0.014) were predictors of higher INR values; on the other hand, lower values of IFN-γ (B = −0.306; *p* = 0.017) were a predictor of higher aPTT values, while male gender (B = −2.771; *p* = 0.008) and confirmed contact with a confirmed case of COVID-19 (B = −2.417; *p* = 0.019) were predictors of higher blood fibrinogen values (Table 3).

### 3.4. Cytokine Profile as a Predictor of Coagulation Status in a Model Controlled for Clinical Characteristics in Patients with COVID-19 Infection

In the Clinical Model, longer duration of symptoms of COVID-19 before hospitalization (B = 69.672; *p* = 0.002) and use of meropenem (B = 1237.220; *p* = 0.014) were predictors of higher D-dimer values; non-use of oxygen therapy (B = −3.077; *p* = 0.018) and corticosteroid use (B = 2.458; *p* = 0.048) were predictors of higher INR values; shorter duration of COVID-19 symptoms before hospitalization (B = −0.538; *p* = 0.042) and arrhythmia (B = 18.874; *p* = 0.001) were predictors of longer prothrombin times; lower IFN-γ values (B = −0.306; *p* = 0.017), non-use of oxygen therapy (B = −6.134; *p* = 0.048), and arrhythmia (B = 10.159; *p* = 0.004) were predictors of aPTT; and shorter duration of COVID-19 symptoms before hospitalization (B = −0.057; *p* = 0.044) and greater lung-field involvement (B = 0.361; *p* = 0.012) were predictors of higher blood fibrinogen values (Table 4).

### 3.5. Cytokine Profile as a Predictor of Coagulation Status in a Model Controlled for Laboratory Parameters in Patients with COVID-19 Infection

In the Laboratory Model, a higher AST value was a predictor of higher INR values (B = 0.034; *p* = 0.006); higher direct bilirubin values (B = 0.278; *p* = 0.013) and potassium on admission (B = 1.234; *p* = 0.007) were predictors of higher blood fibrinogen values; and a lower lymphocyte value was a predictor (B = −2.728; *p* = 0.050) of higher platelet values. Furthermore, higher IL-6 (B = 0.137; *p* = 0.003) values were a predictor of higher prothrombin time; lower IFN-γ values (B = 0.251; *p* = 0.050) predicted higher aPTT; and higher IL-5 values significantly predicted higher INR (B = 0.152; *p* = 0.040) and prothrombin time values (B = 0.412; *p* = 0.043) (Table 5).

## 4. Discussion

The results of our research showed that COVID-19 patients with severe form of disease had higher average values of IL-2, Il-6, IL-17A, and TNF-α when compared to the group with mild/moderate disease. Furthermore, levels of IL-5, IL-6, IL-17F, and IFN-γ significantly correlated with coagulation parameters. Socio-demographic parameters such as lower level of education, gender, and contact with a confirmed case of COVID-19, as well as clinical factors such as duration of COVID-19 symptoms before hospitalization, use of meropenem, involvement of inflamed lung fields, presence of arrhythmia, use of oxygen therapy, and use of corticosteroids were associated with coagulation parameters. Regarding laboratory parameters, values of AST, direct bilirubin and potassium were also associated with coagulation parameters.

Since the beginning of the pandemic, the importance of thrombotic complications for the outcome of COVID-19 disease has been recognized; thromboembolism was recognized as the cause of death in about two thirds of autopsied patients [17,20,21,22,23,24,25]. Longer PT/aPTT time and noticeably higher D-dimer levels are observed in SARS-CoV-2 patients [26,27,28]. An overstimulated immune system and the production of cytokines, chemokines, and other stimuli with accompanying hypoxia that occur in response to SARS-CoV-2 infection cause uncontrolled tissue damage, endothelial activation, and endothelial-cell death [29,30]. Endothelial damage releases a tissue factor that interacts with vascular coagulation factors and initiates the extrinsic coagulation pathway [31]. Furthermore, tissue damage, released nucleic acids, and exposed collagen trigger the intrinsic coagulation pathway by activating factor XII [26]. IL-1β, IL-2, IL-6, IL-8, IL-10, TNF-α, MCP-1, IFN-γ, HGF, and other proinflammatory cytokines are important inflammatory-system mediators that also lead to coagulation disorders [32,33]. The results of the studies published so far suggest that pro-inflammatory cytokines such as IL-1 and IL-6 have a very significant role in the pathogenesis of CAC [34]. These cytokines are thought to mediate thrombosis by activating platelets, endothelium, monocytes, and coagulation factor VIIa [34]. The results of our research are consistent with these findings, since we showed that higher values of IL-6 and IL-5 and lower values of IL-17F and IFN-γ are associated with coagulation disorders; more precisely, they are associated with prolonged coagulation time as measured by INR, aPTT, and prothrombin time in hospitalized patients at admission. A key cytokine that is significantly upregulated in cases of severe COVID-19 infection is IL-6, which acts as a major activator of coagulopathy by promoting tissue-factor expression and enhancing fibrinogen and platelet production by inducing transcription of clotting factors in the liver and of tissue factor in the endothelium [35,36,37]. Generally speaking, IFN-γ, IL-6, and IL-10 are the primary cytokines that are increased in cytokine-storm patients, and proinflammatory cytokines showed a stronger association with coagulation parameters, especially IL-6 [17,38]. Patients with COVID-19 who required mechanical ventilation had an observed high median IL-6 of 121–218 pg/mL, whereas patients who have sepsis from community-acquired pneumonia have a median IL-6 level of 55.6 pg/mL [35,39,40]. The considerable variation in the pattern of coagulopathy in these patients may be explained by the significant change in IL-6 levels, which are most likely directly induced by the COVID-19 infection. It is known that in patients with COVID-19 infection, activation of the Th1 immune response is dominant, while activation of the Th2 immune response is much weaker [41]. Therefore, the production of Th2 pro-inflammatory cytokines, such as IL-5 or IL-9, is much less pronounced in patients with COVID-19 infection [41]. Thus, the results of some studies have shown that in patients with COVID-19 infection, the concentrations of IL-5 and IL-9 are not significantly higher than in healthy controls [41]. In our study, we observed an association of higher IL-5 values with prolonged bleeding time. More recent studies have shown that high levels of IL-5 are associated with better survival in critical patients with COVID-19 [42]. IL-5- and eosinophil-mediated inflammation regulates the expression of genes involved in cell proliferation, survival, and maturation, as well as effector functions of B cells and eosinophils [43]. Because of their prepared granules, which include cytotoxic proteins such as eosinophil peroxidase, major basic protein, eosinophil neurotoxin, and eosinophil cationic protein, as well as reactive nitrogen species, eosinophils are crucial in the host’s defense against viral infections [44]. It is unlikely that eosinophils directly contributed to the favorable outcomes, but activated CD8 T-cells mediate antiviral responses leading to improved survival [42,44]. We also found associations between lower values of IL-17F and IFN-γ and prolonged coagulation time as measured by INR, aPTT, and prothrombin time. IL-17 is a multifunctional cytokine that exhibits a mixed immunopathological effect, ranging from anti-inflammatory to pro-inflammatory, depending on the condition [45]. However, when it comes to COVID-19 infection, it has been shown that IL-17, as well as other cytokines produced by Th17 helper lymphocytes, exert a significant pro-inflammatory effect and that their levels are directly correlated with the severity of the disease [46,47]. However, it has been observed that increased IL-6 levels are a modulator of the differentiation of pro-inflammatory T helper cells that produce IL-17 (Th17) and that it reduces the generation of regulatory T cells (Treg) that play a significant role in suppressing inflammation [48]. Patients with gene polymorphisms that result in lower IL-17 production had higher 30-day survival rates [49]. IL-17 has been observed to be associated with endothelial dysfunction and thrombophilia in patients with COVID-19 via induction of platelet aggregation, activation of endothelial cells, stimulation of tissue-factor production, and modulation of thrombomodulin expression. Activation of coagulation in turn enhances the fibrotic process and increases lung fibrosis; thus, inhibition of IL-17 contributes to the prevention of fibrosis in patients with COVID-19 by interfering with coagulation pathways [47]. IFN-γ is a key modulator of immunity against intracellular microorganisms and offers protection against specific viral infections [50]. It has been observed that in patients with severe COVID-19, the IFN-γ response is usually either reduced or delayed [51]. An inverse correlation was observed between a levels of IL-6 and IFN-γ in plasma, and a low level of IFN-γ in the initial phase of COVID-19 was associated with hospitalization [52]. As our patients’ cytokines were measured on admission, it is possible that the negative association of IFN-γ with prolonged bleeding time is a consequence of the delayed production of this cytokine.

The results of our study may have implications for the choice of therapy in the treatment of critically ill patients. Tocilizumab is already known to be effective in treating critically ill patients with COVID-19, reducing all-cause mortality, the frequency of use of mechanical ventilation, and the length of stay of hospitalized patients with COVID-19 [53]. Patients in the first stage of COVID-19, which is marked by limited inflammation and viral replication, should not receive anti-IL-5 drugs because eosinophils are involved in the antiviral immune response. However, high-risk hypoxic patients in the second stage of the disease may be the best candidates in whom to test the use of biological drugs against IL-5 and prevention of eosinophil recruitment [44].This hypothesis is supported by the findings of Lucas et al. (2020), who observed that eosinophil counts increased 11–15 days after symptom onset and that IL-5 levels increased within 6–10 days after symptom onset [54]. Similar clinical circumstances exist for dexamethasone’s effect on COVID-19 mortality, which may also be brought on by eosinophil apoptosis [55]. A recent study found that the IL-17 inhibitor ixekizumab, the indirect IL-6 inhibitor colchicine, and IL-2 to be safe but ineffective for treating COVID-19 [56]. Surprisingly, the anti-IL-17 drugs netacimab and secukinumab reduce the risk of pulmonary embolism in patients with severe COVID-19, indicating that IL-17 may be a major factor causing this complication of COVID-19, given its documented prothrombotic and procoagulant effects [57,58]. Additionally, because IFN-γ is a modulator of immunity against intracellular microorganisms, recombinant (r)IFN-γ therapy has been shown to offer protection against certain viral pathogens, to be well tolerated, and to shorten time to hospital discharge [59].

Our study demonstrated the association of male gender and contact with a confirmed case of COVID-19 with higher blood fibrinogen. Since the beginning of the pandemic, it has been known that male gender is a risk factor for the emergence of a severe form of COVID-19, while close contact with an infected person carries a higher risk of infection with a higher viral load and consequent worsening of COVID-19 [60,61]. On the other hand, higher d-dimer levels are associated with levels of inflammatory markers such as C-reactive protein and a worse prognosis for COVID-19 due to a higher risk of activation of thrombotic factors [62]. According to our research, greater d-dimer values were linked to both the use of meropenem and longer symptom duration prior to hospitalization. According to recent research, patients who experience persistent symptoms of SARS-CoV-2 are likely to develop microthrombi, which is something that the ultrasonography of their leg veins was unable to detect [63,64]. An increase in D dimer levels is associated with viral infection, inflammatory factors, and longer duration of COVID symptoms [65]. Antibiotics were found to increase the risk of acute organ injury in hospitalized patients with COVID-19, and intravenous moxifloxacin and meropenem increased mortality in patients with suspected bacterial infection as well as in patients without evidence of bacterial infection [66]. Elevated D-dimer levels have been reported to be significantly correlated with diabetes mellitus and advanced age as well [67].

Shorter duration of COVID-19 symptoms before hospitalization and greater involvement of lung fields were associated with higher values of fibrinogen in our patients, which indicates a rapid development of the disease with more extensive inflammation and rapid progression. Also in our study, an association between not using oxygen therapy with a higher INR was observed, while the use of corticosteroids was associated with a higher INR and a long aPTT. The study by Rauch et al. in which coagulation biomarkers as independent predictors of increased oxygen requirements in COVID-19 were examined has already established that in COVID-19 patients, increased levels of fibrinogen are significantly associated with the risk of increased oxygen requirements [68]. Furthermore, previous studies have concluded that higher fibrinogen levels are generally observed in critically ill patients, especially those with hypoxemia reflecting inflammation [69]. Regarding the use of corticosteroids, recent studies have shown that the use of dexamethasone is associated with a reduction in proinflammatory and procoagulant profiles in critically ill patients with COVID-19 [70]. A retrospective review showed an increase in international normalized ratio (INR) in the majority of patients for whom corticosteroids were added to warfarin therapy [71].

The main limitation of our study is the cross-sectional design because it does not allow us to draw conclusions about causal associations between cytokine levels and coagulation abnormalities. Therefore, in future studies, a longitudinal design would be more appropriate so that changes in the investigated parameters could be monitored over time. Since this was a single-center study, future studies should focus on including more patients from multiple centers to avoid potential bias and thus increase the generalizability of the findings. Additionally, only hospitalized patients who did not have asymptomatic or mild forms of COVID-19 were included in our study. The inclusion of these groups of patients in subsequent studies could show greater differences in the investigated parameters and their correlations. Although our study controlled for a number of variables, there is the possibility of unmeasured confounding factors whose future inclusion in the analysis could affect the results of future studies. For example, a recent study revealed an association between the human cathelicidin antimicrobial peptide LL37, which has antibacterial and anti-biofilm activity and immunomodulatory function, and abnormal blood clotting in patients with COVID-19 [72]. Levels of the cathelicidin peptide LL-37 increase the activity of coagulation factors such as factor Xa (FXa) and thrombin, which can cause hypercoagulation. Also, cathelicidin peptide LL-37 showed a negative correlation with thrombin time but a positive correlation with fibrinogen level. While deletion of cathelicidin prevented thrombosis in vivo, injection of cathelicidin peptide enhanced the occurrence of thrombosis. By stimulating coagulation factors, elevated levels of the antimicrobial peptide cathelicidin (LL-37) during SARS-CoV-2 infection can cause hypercoagulation in patients with COVID-19 [72].

## 5. Conclusions

In conclusion, our results indicate a significant correlation between pro-inflammatory cytokines and coagulation-related parameters. COVID-19 patients with severe form of disease had higher values of IL-2, IL-6, IL-17A, and TNF-α. Furthermore, the proinflammatory cytokines IL-5, IL-6, IL-17F, and IFN-γ were negatively associated with coagulation parameters. Regression analyses in the Socio-Demographic Model showed that lower concentrations of IL-17F were a predictor of higher INR values, while lower values of IFN-γ significantly predicted higher values of aPTT, as well as non-use of oxygen therapy and arrhythmia. In the Laboratory Model, our study has shown that higher IL-6 levels were associated with prothrombin time, while IFN-γ was associated with aPTT and IL-5 was associated with INR values and prothrombin times. Additionally, a lower level of education, non-use of oxygen therapy, corticosteroid use, and higher AST values were predictors of higher INR values. Furthermore, shorter duration of COVID-19 symptoms before hospitalization predicted longer prothrombin times, while longer duration of symptoms before hospitalization and use of meropenem predicted higher D-dimer values. Male gender, confirmed contact with a confirmed case of COVID-19, shorter duration of COVID-19 symptoms before hospitalization, greater lung-field involvement, higher direct bilirubin values, and higher potassium levels on admission were predictors of higher blood fibrinogen values.

Disorders of the coagulation cascade and blood hypercoagulability are important aspects of the pathogenesis of COVID-19 infection. Therefore, laboratory parameters that indicate disorders in the blood-coagulation process, particularly D-dimer and fibrinogen levels, are important prognostic predictors in COVID-19 patients. The results of our study indicated that the values of the coagulation profile are disturbed by pro-inflammatory cytokines. However, more high-quality research is required to better understand the pathophysiology of SARS-CoV-2, to further develop a cure, and to better manage possible thromboembolic complications. Furthermore, in the future, the combination of other cytokine blockers in the treatment of COVID-19 should be considered, as should their combination with anticoagulant drugs while analyzing potential interactions between drugs to reduce the number of complications in these patients.

## Figures and Tables

**Table 1:** Differences in socio-demographic characteristics and their correlation with coagulation status in patients with COVID-19 infection hospitalized at the Kosovska Mitrovica Health Center.

Socio-Demographic Variables	D-Dimer (<500 ng/mL)	INR	Prothrombin Time (s)	aPTT (s)	Fibrinogen (2.0–5.0 g/L)	Platelets (150–450 × 10^9^/L)
Age (r)	* 0.240	0.125	0.276	0.242	0.067	−0.049
Gender						
Male	668.48 ± 1448.36	2.25 ± 2.60	16.29 ± 9.59	28.64 ± 3.84	** 6.00 ± 1.49	229.86 ± 120.12
Female	489.12 ± 527.88	1.26 ± 0.30	13.60 ± 3.30	30.50 ± 7.53	** 4.98 ± 1.58	217.47 ± 83.22
BMI (r)	0.024	0.263	0.202	−0.297	0.143	−0.095
Change in BMI over 6 months						
Decrease of BMI	727.39 ± 1068.14	1.27 ± 0.43	13.97 ± 4.64	29.22 ± 3.65	5.37 ± 1.43	253.43 ± 138.97
Same BMI	595.71 ± 1574.03	2.02 ± 2.06	18.29 ± 11.61	29.28 ± 6.92	6.13 ± 1.55	212.07 ± 85.58
Increase of BMI	359.31 ± 203.38	3.61 ± 4.38	12.26 ± 0.73	29.03 ± 4.74	5.36 ± 1.82	188.80 ± 49.17
Relationship status						
Single	683.05 ± 1579.68	2.15 ± 2.04	* 19.69 ± 11.75	30.21 ± 6.82	6.09 ± 1.68	221.70 ± 92.62
In relationship/marriage	559.48 ± 902.2	1.83 ± 2.41	* 12.48 ± 0.91	28.52 ± 3.74	5.43 ± 1.46	229.28 ± 121.64
Level of education						
<8 years	* 1498.00 ± 2995.62	** 1.67 ± 0.48	17.75 ± 6.01	38.90 ± 13.15	5.64 ± 1.54	261.31 ± 136.78
8–12 years	* 411.00 ± 395.40	** 1.22 ± 0.36	13.39 ± 3.87	28.37 ± 4.11	5.78 ± 1.61	224.81 ± 118.11
>12 years	* 709.57 ± 1245.31	** 3.67 ± 3.70	19.72 ± 13.61	28.86 ± 3.12	5.59 ± 1.61	210.15 ± 66.50
Employment						
Unemployed	364.40 ± 206.09	1.45 ± 0.49	15.87 ± 5.31	37.30 ± 15.41	5.13 ± 2.04	265.18 ± 116.72
Employed	524.85 ± 834.70	2.24 ± 2.83	14.34 ± 7.22	28.37 ± 4.01	5.51 ± 1.56	215.36 ± 106.57
Pensioner	819.75 ± 1796.45	1.61 ± 1.00	17.50 ± 10.83	29.12 ± 3.54	6.24 ± 1.34	227.04 ± 111.86
Smoking status						
Non-smoker	* 487.00 ± 717.97	* 2.31 ± 2.58	* 16.81 ± 9.58	29.36 ± 5.78	5.77 ± 1.67	212.86 ± 83.39
Ex-smoker	* 1108.90 ± 2245.89	* 1.16 ± 0.05	* 12.73 ± 0.55	28.44 ± 3.55	5.78 ± 1.25	255.06 ± 137.20
Smoker	* 268.17 ± 102.14	* 1.04 ± 0.07	* 11.47 ± 0.75	29.60 ± 0.85	5.16 ± 1.55	282.10 ± 216.05
Smoking history in years (r)	−0.352	−0.556	−0.556	0.116	0.006	0.118
Number of cigarettes per day (r)	0.287	−0.707	−0.707	0.530	0.032	0.119
Alcohol consumption						
Never	415.56 ± 328.87	1.76 ± 1.98	15.55 ± 8.77	30.42 ± 6.58	5.37 ± 1.31	230.25 ± 94.10
Monthly and less often	609.00 ± 974.27	2.35 ± 3.06	15.77 ± 9.50	26.99 ± 3.45	5.72 ± 1.89	213.61 ± 96.68
Weekly	1017.07 ± 2492.03	1.72 ± 0.99	15.01 ± 6.24	30.34 ± 4.02	6.18 ± 1.10	252.48 ± 158.76
Time to go to sleep (r)	−0.140	−0.340	−0.340	−0.046	−0.106	−0.140
Sleep length during the night in hours (r)	0.140	0.084	0.019	−0.227	0.091	−0.112
Length of sleep during vs. before COVID-19 disease						
Less	482.00 ± 701.18	1.36 ± 0.47	14.92 ± 5.06	29.37 ± 6.39	5.51 ± 1.65	231.26 ± 117.01
No change	747.50 ± 1891.90	2.41 ± 2.59	20.20 ± 14.52	28.33 ± 4.45	6.17 ± 1.58	203.53 ± 74.86
More	684.49 ± 1055.38	2.38 ± 3.19	12.52 ± 1.02	29.55 ± 4.22	5.50 ± 1.41	243.84 ± 130.03
Quality of sleep before COVID-19 disease						
Bad/very bad	502.43 ± 216.92	2.04 ± 0.89	22.30 ± 9.62	30.75 ± 5.73	5.90 ± 1.19	256.36 ± 102.79
Average	559.33 ± 756.67	2.32 ± 2.84	16.57 ± 10.15	29.66 ± 6.21	5.75 ± 1.63	237.43 ± 113.98
Good/very good	673.04 ± 1590.89	1.37 ± 0.74	12.53 ± 0.98	28.29 ± 3.56	5.65 ± 1.63	213.03 ± 107.97
Quality of sleep during COVID-19 disease						
Bad/very bad	375.41 ± 194.65	* 1.47 ± 0.59	** 16.15 ± 6.44	28.73 ± 3.81	5.42 ± 1.31	214.37 ± 91.54
Average	591.45 ± 741.62	* 2.67 ± 3.15	** 17.91 ± 11.21	29.99 ± 7.28	5.98 ± 1.47	234.15 ± 114.97
Good/very good	759.41 ± 1841.08	* 1.33 ± 0.75	** 12.09 ± 0.93	28.86 ± 4.07	5.65 ± 1.78	222.77 ± 114.69
Use of supplements to strengthen immunity						
No	638.69 ± 1411.03	2.15 ± 2.53	* 16.65 ± 9.40	29.63 ± 5.53	5.85 ± 1.60	217.41 ± 91.79
Yes	573.94 ± 912.59	1.42 ± 0.87	* 12.23 ± 0.39	27.91 ± 4.02	5.41 ± 1.52	243.02 ± 138.69
Length of supplement use in the days before infection with COVID-19 (r)	0.292	0.342	0.000	0.018	0.206	0.389 *
Close contact with a confirmed case of COVID-19						
No	445.70 ± 423.59	1.94 ± 1.94	17.92 ± 11.33	28.04 ± 3.74	** 6.19 ± 1.54	225.61 ± 124.68
Unknown	789.88 ± 1803.26	2.12 ± 3.02	13.26 ± 3.01	30.20 ± 7.13	** 5.34 ± 1.64	235.45 ± 105.29
Yes	730.69 ± 1381.43	1.69 ± 1.06	13.20 ± 1.12	30.12 ± 3.39	** 4.92 ± 1.03	202.57 ± 61.47
Visit to a COVID-19 facility						
No	587.19 ± 1284.16	2.18 ± 2.50	16.18 ± 9.35	28.91 ± 5.62	* 5.89 ± 1.52	226.04 ± 114.05
Yes	707.61 ± 1132.20	1.20 ± 0.09	13.19 ± 0.95	30.40 ± 3.12	* 4.82 ± 1.59	226.31 ± 96.72
A family member had COVID-19						
No	582.94 ± 1208.28	1.99 ± 2.36	15.89 ± 8.82	29.11 ± 5.58	5.74 ± 1.61	222.52 ± 112.31
Yes	860.80 ± 1555.60	1.79 ± 1.20	12.90 ± 1.86	29.90 ± 1.25	5.12 ± 0.92	255.94 ± 86.57

INR—international normalized ratio; aPTT—activated partial thromboplastin time; BMI—body mass index; r—correlation coefficient; *p*—statistical significance; bold values indicate statistical significance; * *p* < 0.05; ** *p* < 0.01; (Chi-square, Mann–Whitney U test).

**Table 2:** The difference in the concentration of cytokines in patients with COVID-19 infection hospitalized in the Kosovska Mitrovica Health Center in relation to the severity of the clinical manifestations.

Cytokines (pg/mL)	Reference Range (pg/mL)	Mild/Moderate (*n* = 70) Mean ± SD	Severe (*n* = 43) Mean ± SD	*p*
**IL-2**	0–4.18 [20]	54.35 ± 5.27	74.13 ± 14.68	**0.001**
IL-4	0–2.13 [20]	471.95 ± 13.50	475.35 ± 15.56	0.292
IL-5	0–2.13 [20]	142.71 ± 7.79	144.26 ± 11.82	0.635
**IL-6**	<7 [17]	101.67 ± 19.12	157.49 ± 56.33	**0.001**
IL-9	0.52–1.92 [21]	88.28 ± 6.12	87.93 ± 4.19	0.909
IL-10	<9.1 [17]	355.22 ± 16.95	356.88 ± 14.50	0.526
IL-13	9.17–22.59 [22]	10.53 ± 0.35	10.71 ± 0.54	0.131
**IL-17A**	0–20.27 [20]	7.41 ± 1.60	9.48 ± 3.24	**0.001**
IL-17F	0.17–5.92 [23]	763.19 ± 39.59	774.33 ± 44.90	0.215
IL-21	<4.6 [24]	289.91 ± 14.45	284.82 ± 18.95	0.177
IL-22	<7.8 [24]	6.73 ± 0.17	6.72 ± 0.18	0.735
IFN-γ	0–2.68 [20]	108.12 ± 14.38	109.75 ± 4.45	0.152
**TNF-α**	<8.1 [17]	30.63 ± 19.45	50.00 ± 16.00	**0.001**

IL—interleukin; IFN-γ—interferon gamma; TNF-α—tumor necrosis factor alpha; SD—standard deviation; *p* statistical significance; bolded values are statistically significant (Mann–Whitney U test). Reference range of serum cytokines in healthy individuals (without COVID-19) taken from a literature survey.

**Table 3:** Regression analysis of cytokine profile as a predictor of coagulation status in a model controlled for socio-demographic characteristics in patients with COVID-19 infection hospitalized in the Kosovska Mitrovica Health Center.

Socio-Demographic Model	D-Dimer (<500 ng/mL)		INR		Prothrombin Time (s)		aPTT (s)		Fibrinogen (2.0–5.0 g/L)		Platelets (150–450 × 10^9^/L)	
	B	*p*	B	*p*	B	*p*	B	*p*	B	*p*	B	*p*
Constant	2183.100	0.560	5.878	0.568	62.349	0.130	76.022	0.003	−1.069	0.816	155.872	0.513
IL-5 (pg/mL)	−9.569	0.535	−0.108	0.143	−0.175	0.538	0.062	0.672	−0.003	0.871	−1.380	0.239
IL-6 (pg/mL)	−2.737	0.372	−0.001	0.852	0.013	0.634	−0.015	0.713	0.005	0.360	0.272	0.217
**IL-17F** (pg/mL)	0.139	0.971	**0.024**	**0.050**	−0.004	0.938	−0.027	0.273	0.006	0.233	0.292	0.275
**IFN-γ** (pg/mL)	0.413	0.988	−0.056	0.320	−0.187	0.397	**−0.306**	**0.017**	0.018	0.619	0.069	0.972
Constant	1487.863	0.762	−7.035	0.614	40.040	0.439	91.942	0.011	0.588	0.559	241.476	0.427
IL-5 (pg/mL)	−4.142	0.807	−0.157	0.088	−0.163	0.618	−0.005	0.981	0.067	0.947	−1.664	0.176
IL-6 (pg/mL)	−3.106	0.340	0.001	0.944	0.011	0.696	−0.022	0.678	1.247	0.218	0.350	0.130
**IL-17F** (pg/mL)	1.081	0.812	**0.035**	**0.030**	0.017	0.763	−0.016	0.585	0.935	0.354	0.288	0.334
**IFN-γ** (pg/mL)	−12.020	0.678	−0.054	0.340	−0.226	0.281	**−0.347**	**0.017**	−0.051	0.960	0.224	0.912
Gender	−279.922	0.413	0.982	0.444	−1.824	0.699	−2.232	0.405	**−2.771**	**0.008**	−3.728	0.878
Age (r)	15.510	0.294	0.070	0.136	0.212	0.218	0.013	0.885	0.402	0.690	−0.588	0.553
Relationship status	−204.873	0.544	0.142	0.892	−5.947	0.134	−2.622	0.271	−1.119	0.268	−8.268	0.747
Level of education	−132.442	0.644	**2.948**	**0.014**	7.202	0.087	−3.474	0.167	−0.025	0.980	−21.233	0.273
Smoking status	187.908	0.448	−0.129	0.851	−1.071	0.673	−0.662	0.726	0.215	0.831	31.206	0.082
Quality of sleep during COVID-19	210.542	0.303	−0.902	0.203	−3.027	0.245	1.713	0.281	0.260	0.796	7.149	0.617
Use of supplements to strengthen immunity	−60.805	0.858	0.428	0.700	1.867	0.650	−3.005	0.247	0.166	0.869	28.584	0.249
Close contact with a confirmed case of COVID-19	157.137	0.456	0.848	0.252	−0.080	0.976	1.634	0.344	**−2.417**	**0.019**	−5.934	0.704
Visit to a COVID-19 facility	190.285	0.634	−1.791	0.151	−2.234	0.618	2.491	0.382	−1.326	0.190	−13.589	0.630

IL—interleukin; IFN-γ—interferon gamma; INR—international normalized ratio; aPTT—activated partial thromboplastin time; r—correlation coefficient; B—unstandardized regression coefficient, *p*—statistical significance; bold values indicate statistical significance.

**Table 4:** Regression analysis of cytokine profile as a predictor of coagulation status in a model controlled for clinical characteristics in patients with COVID-19 infection hospitalized in the Kosovska Mitrovica Health Center.

“Clinical” Model	D-Dimer (<500 ng/mL)		INR		Prothrombin Time (s)		aPTT (s)		Fibrinogen (2.0–5.0 g/L)		Platelets (150–450 × 10^9^/L)	
	B	*p*	B	*p*	B	*p*	B	*p*	B	*p*	B	*p*
Constant	2183.100	0.560	5.878	0.568	62.349	0.130	76.022	0.003	−1.069	0.816	155.872	0.513
IL-5 (pg/mL)	−9.569	0.535	−0.108	0.143	−0.175	0.538	0.062	0.672	−0.003	0.871	−1.380	0.239
IL-6 (pg/mL)	−2.737	0.372	−0.001	0.852	0.013	0.634	−0.015	0.713	0.005	0.360	0.272	0.217
IL-17F (pg/mL)	0.139	0.971	0.024	0.054	−0.004	0.938	−0.027	0.273	0.006	0.233	0.292	0.275
**IFN-γ** (pg/mL)	0.413	0.988	−0.056	0.320	−0.187	0.397	**−0.306**	**0.017**	0.018	0.619	0.069	0.972
Constant	3973.542	0.287	−1.398	0.913	8.276	0.832	49.924	0.055	−0.636	0.900	77.286	0.763
IL-5 (pg/mL)	−17.815	0.227	−0.058	0.440	−0.018	0.936	0.058	0.654	−0.007	0.699	−1.170	0.328
IL-6 (pg/mL)	−5.254	0.135	0.009	0.429	0.028	0.409	0.090	0.110	0.001	0.906	0.376	0.171
IL-17F (pg/mL)	−2.450	0.516	0.031	0.086	0.046	0.383	−0.007	0.813	0.002	0.646	0.247	0.371
**IFN-γ** (pg/mL)	10.003	0.695	−0.113	0.125	−0.267	0.227	**−0.296**	**0.035**	0.040	0.247	0.398	0.845
**Duration of symptoms before hospitalization**	**69.672**	**0.002**	−0.010	0.899	**−0.538**	**0.042**	−0.148	0.347	**−0.057**	**0.044**	2.436	0.139
**Oxygen therapy**	−380.914	0.250	**−3.077**	**0.018**	−6.744	0.077	**−6.134**	**0.048**	0.130	0.824	−40.313	0.143
**Affected lung fields**	27.976	0.748	−0.128	0.746	0.891	0.459	0.628	0.418	**0.361**	**0.012**	6.938	0.321
**ECG-arrhythmia**	12.261	0.980	2.287	0.135	**18.874**	**0.001**	**10.159**	**0.004**	−0.214	0.740	−19.914	0.604
**Corticosteroids**	58.684	0.845	**2.458**	**0.048**	7.176	0.056	1.779	0.467	0.529	0.239	11.078	0.642
Favipiravir	−54.180	0.863	−0.445	0.670	−2.756	0.389	−1.248	0.611	−0.127	0.766	−0.192	0.994
Vancomycin	−112.112	0.854	0.121	0.958	1.562	0.823	0.190	0.961	1.090	0.157	42.330	0.358
**Meropenem**	**1237.220**	**0.023**	−0.602	0.767	−1.344	0.828	−3.819	0.266	−0.648	0.311	7.809	0.845

IL—interleukin; IFN-γ—interferon gamma; INR—international normalized ratio; aPTT—activated partial thromboplastin time; ECG—electrocardiogram; B—unstandardized regression coefficient; *p*—statistical significance; bold values indicate statistical significance.

**Table 5:** Regression analysis of cytokine profile as a predictor of coagulation status in a model controlled for laboratory parameters in patients with COVID-19 infection hospitalized in the Health Center of Kosovska Mitrovica.

“Laboratory” Model	D-Dimer (<500 ng/mL)		INR		Prothrombin Time (s)		aPTT (s)		Fibrinogen (2.0–5.0 g/L)		Platelets (150–450 × 10^9^/L)	
	B	*p*	B	*p*	B	*p*	B	*p*	B	*p*	B	*p*
Constant	3803.486	0.439	12.360	0.238	107.240	0.001	100.710	0.001	−1.552	0.754	135.329	0.655
IL-5 (pg/mL)	−6.268	0.750	**0.152**	**0.040**	**0.412**	**0.043**	−0.072	0.663	−0.004	0.835	−1.706	0.204
IL-6 (pg/mL)	−6.573	0.224	0.001	0.937	**0.137**	**0.003**	0.018	0.684	0.009	0.153	−0.336	0.278
IL-17F (pg/mL)	−1.603	0.767	0.018	0.119	−0.064	0.051	−0.046	0.092	0.005	0.418	0.443	0.190
**IFN-γ** (pg/mL)	−1.688	0.962	−0.027	0.604	−0.001	0.995	**−0.251**	**0.050**	0.030	0.432	0.330	0.891
Constant	−4278.772	0.706	20.820	0.297	162.756	0.101	55.273	0.437	6.713	0.504	646.970	0.230
IL-5 (pg/mL)	17.324	0.424	−0.070	0.355	−0.396	0.279	−0.191	0.482	0.025	0.180	−0.877	0.515
IL-6 (pg/mL)	−7.356	0.257	0.014	0.389	0.099	0.207	0.022	0.724	0.006	0.361	−0.425	0.215
IL-17F (pg/mL)	−1.453	0.820	0.016	0.260	−0.025	0.706	−0.081	0.187	−0.002	0.732	0.369	0.278
**IFN-γ** (pg/mL)	−9.320	0.792	−0.002	0.959	0.062	0.758	−0.249	0.134	0.003	0.915	0.130	0.954
C-reactive protein (<5 mg/L)	−5.641	0.125	−0.015	0.067	0.017	0.647	0.049	0.295	0.006	0.063	−0.220	0.299
Sedimentation (mm/h)	9.250	0.282	0.001	0.957	−0.066	0.404	−0.062	0.444	0.006	0.466	0.493	0.315
WBC (3.71–10.67 × 10^9^/L)	42.254	0.460	0.033	0.883	−0.608	0.574	−0.140	0.878	−0.040	0.435	3.314	0.136
Lymphocytes (18.94–46.71%)	−23.920	0.452	0.001	0.988	0.183	0.403	0.183	0.268	−0.023	0.385	**−2.728**	**0.050**
Albumins (41–51 g/L)	−69.749	0.227	0.051	0.652	−0.466	0.392	0.224	0.581	−0.010	0.833	−6.195	0.051
Alkaline phosphatase (30–120 U/L)	−1.118	0.892	−0.022	0.269	−0.035	0.696	0.099	0.147	0.001	0.904	0.821	0.118
**Direct bilirubin** (<3.4 µmol/L)	17.086	0.884	−0.387	0.297	−1.289	0.460	−0.038	0.979	**0.278**	**0.013**	−4.738	0.472
Cholesterol (<5.2 mmol/L)	334.242	0.212	−0.207	0.699	1.184	0.643	1.115	0.581	−0.170	0.555	−0.421	0.978
**AST** (35–50 U/L)	3.206	0.610	**0.034**	**0.006**	0.025	0.609	0.013	0.782	0.011	0.070	−0.293	0.420
Urea (2.8–7.2 mmol/L)	31.668	0.630	0.257	0.172	1.266	0.159	−0.714	0.347	−0.040	0.543	−2.233	0.578
Na^+^ (135–147 mmol/L)	24.666	0.729	−0.146	0.322	−0.584	0.401	0.554	0.341	−0.070	0.232	−2.117	0.566
**K^+^** (4.5–5.4 mmol/L)	762.492	0.091	−0.884	0.274	0.331	0.929	−1.943	0.491	**1.234**	**0.007**	−0.518	0.984

IL—interleukin; IFN-γ—interferon gamma; INR—international normalized ratio; aPTT—activated partial thromboplastin time; WBC—white blood cells; AST—aspartate aminotransferase; Na^+^—sodium; K^+^—potassium; B—unstandardized regression coefficient, *p*—statistical significance; bold values indicate statistical significance.

## Data Availability

Data is unavailable due to privacy and ethical restrictions.

## Supplementary Materials

The following supporting information can be downloaded at: https://www.mdpi.com/article/10.3390/biomedicines12061281/s1, Table S1: The difference in clinical characteristics and applied therapy, as well as their correlation with coagulation status in patients with COVID-19 infection hospitalized in the Kosovska Mitrovica Health Center; Table S2: Correlation of laboratory parameters with coagulation status in patients with COVID-19 infection hospitalized at the Kosovska Mitrovica Health Center; Table S3: Correlation of cytokine concentrations with coagulation status in patients with COVID-19 infection hospitalized in the Kosovska Mitrovica Health Center.

## Abbreviations

ACE2	angiotensin-converting enzyme 2
ARDS	acute respiratory distress syndrome
IL	interleukin
TNF-α	tumor necrosis factor α
IFN-γ	interferon-gamma
CAC	COVID-19 associated coagulopathy
PT	prothrombin time
aPTT	activated partial thromboplastin time
INR	international normalized ratio
BMI	body mass index
CT	computed tomography
ECG	electrocardiogram
HDL	high-density lipoprotein
LDL	low-density lipoprotein
AST	aspartate aminotransferase
ALT	alanine transaminase
GGT	gamma-glutamyl transpeptidase
CRP	C-reactive protein
SD	standard deviation
Th2	CD4+ T helper cells
Th1	CD8+ T cytotoxic cells
Treg	regulatory T cells
