# Supplementary material for: The Impact of Cytokines on Coagulation Profile in COVID-19 Patients: Controlled for Socio-Demographic, Clinical, and Laboratory Parameters
Source: Biomedicines. 2024 Jun 10;12(6):1281. doi: 10.3390/biomedicines12061281 (PMC11201770; doi:10.3390/biomedicines12061281)
Supplement: Supplementary file 1 [file biomedicines-12-01281-s001.zip › biomedicines-2952175-supplementary.pdf]

Supplementary Materials:

Supplementary Table S1. The difference in clinical characteristics and applied therapy, as well as their correlation with coagulation status in patients with COVID-19 infection hospitalized in the Kosovska Mitrovica Health Center.

Clinical parameters	D-dimer (<500 ng/ml)	INR	Prothrombin time (s)	aPTT (s)	Fibrinogen (2.0–5.0 g/L)	Platelets (150–450 × 10^9/L)
**Number of present symptoms of COVID-19 per patient**						
No symptoms	368.50±150.16	2.46±2.57	20.75±14.21	27.53±4.53	6.57±1.58	211.75±91.82
One	286.70±102.55	1.12±0.13	12.28±1.39	27.23±3.34	5.60±0.82	175.42±71.34
Two	1773.91±3090.29	1.32±0.39	14.52±4.31	32.54±8.76	4.80±1.25	249.01±126.52
Three	615.23±1009.29	1.16±0.00	12.70±0.00	26.40±0.00	5.87±1.83	256.75±162.13
Four	595.73±686.52	1.18±0.21	12.95±2.33	27.70±4.67	5.22±1.82	189.19±63.77
Five and more	417.87±347.26	2.41±3.03	14.04±5.02	29.88±4.30	5.54±1.58	243.99±103.57
**Duration of symptoms before hospitalization (r)**	0.027	0.029	0.023	0.213	***-0.269**	***0.214**
**Number of chronic diseases per patient**						
No chronic disease	***517.45±872.23**	2.88±3.26	17.31±11.73	28.42±4.53	5.65±1.68	223.21±70.89
One	***1000.61±2104.58**	1.07±0.02	11.70±0.28	25.55±4.31	5.71±1.23	247.94±155.85
Two	***348.00±228.16**	1.27±0.27	13.95±2.98	30.77±6.98	5.48±1.58	216.12±119.18
Three and more	***600.74±540.03**	1.46±0.68	15.78±7.47	29.98±3.18	6.47±2.20	199.08±68.50
**Oxygen therapy**						
No	777.30±1607.19	2.25±2.68	15.40±7.79	30.32±5.91	***5.33±1.63**	226.20±109.55
Yes	384.78±170.14	1.43±0.88	15.71±9.54	27.13±2.60	***6.25±1.35**	225.94±111.92
**Oxygen therapy, number of days (r)**	0.077	-0.370	-0.370	-0.296	-0.255	-0.184
**Oxygen therapy, number of liters (r)**	0.192	-0.176	-0.176	0.272	0.143	-0.157
**High-flow oxygen therapy**						
No	619.88±1275.18	1.99±2.26	15.59±8.42	29.22±5.19	5.69±1.57	224.91±107.68
Yes	511.00±104.65	1.17±0.00	12.90±0.00	/	6.19±2.39	251.46±166.43
**Respirator**						
No	622.59±1282.23	1.99±2.26	15.59±8.42	29.22±5.19	5.68±1.59	223.64±108.40
Yes	488.40±199.36	1.17±0.00	12.90±0.00	/	6.26±1.50	269.60±139.54
**Duration of hospitalization, number of days (r)**	0.183	-0.066	-0.015	-0.332	0.198	-0.078
**COVID-19 severity**						
Mild/moderate	752.40±1581.08	2.25±2.68	15.40±7.79	30.32±5.91	5.40±1.63	228.03±115.74
Severe/very severe	400.50±161.60	1.43±0.88	15.71±9.54	27.13±2.60	6.21±1.38	222.76±100.61
**Outcome**						
Full recovery	627.18±1289.54	1.92±2.27	14.60±6.56	/	5.65±1.59	215.58±86.89
Death	514.25±208.48	2.62±2.05	28.60±22.20	/	6.32±1.49	307.36±177.98

Transfer to other facility	318.00±155.56	/	/	/	5.98±0.00	191.00±37.05
Dismissal at personal request	/	/	/	/	7.48±0.00	800.00±0.00
**X-ray findings on admission**						
No disease	1400.50±1583.21	0.96±0.00	10.60±0.00	30.20±0.00	5.50±0.00	229.03±38.10
Bilateral pneumonia	623.91±1346.15	2.02±2.30	15.78±8.51	29.23±5.25	5.75±1.68	230.97±116.84
Pneumonia of the right lung	476.50±207.60	1.14±0.00	12.50±0.00	23.50±0.00	6.21±0.92	205.48±84.46
Pneumonia of the left lung	392.00±276.83	/	/	31.45±7.14	4.58±1.13	197.53±75.05
**Change in X-ray at discharge**						
No disease/regression	689.48±1409.46	2.06±2.48	14.90±7.16	29.67±5.70	5.57±1.72	222.64±99.53
Stationary finding	341.44±162.82	1.62±1.09	17.60±11.87	28.03±3.26	5.99±1.30	237.43±147.27
Progression	459.00±281.91	/	/	26.40±0.00	5.98±0.70	232.04±99.73
**Affected lung fields (X-ray)**						
No disease	1003.33±1313.97	0.96±0.00	10.60±0.00	30.20±0.00	**4.49±1.02	210.08±53.99
Upper	318.80±67.13	1.06±0.00	11.60±0.00	21.70±0.00	**6.53±0.87	233.2±98.50
Lower	588.17±1501.77	1.86±2.58	13.01±1.13	28.14±3.14	**5.16±1.58	214.33±90.92
Midlle	264.00±0.00	2.34±0.47	25.55±5.02	41.50±9.48	**4.28±1.73	146.73±30.06
Upper and middle	1082.83±1661.43	1.74±1.30	19.04±14.14	26.73±3.31	**6.22±1.26	271.51±181.80
Lower and middle	444.14±354.31	2.50±2.71	16.71±11.64	31.04±4.05	**6.45±1.44	236.76±102.57
All lung fields	714.00±0.00	/	/	/	**8.11±0.00	145.10±0.00
**Auscultatory findings on the lungs**						
Normal	332.43±178.40	3.24±4.02	12.67±1.64	29.62±3.22	5.50±1.58	180.02±53.38
Bilateral cracks	613.87±1456.28	1.45±0.83	15.85±8.97	27.97±4.19	5.87±1.31	242.33±132.05
Unilateral cracks	400.50±164.17	1.16±0.04	12.80±0.42	28.08±5.76	5.81±1.58	237.61±101.66
Weakened breath sounds	1029.17±1684.32	2.33±2.65	18.49±11.42	32.28±7.88	5.61±2.24	207.82±72.65
Harsh breathing sound	810.17±935.65	1.08±0.00	11.90±0.00	/	5.22±1.57	230.73±91.79
**ECG**						
Sinus rhythm	624.87±1274.00	**1.88±2.35	***13.73±5.95	**28.20±3.65	5.72±1.58	227.04±112.50
Arrhythmia	395.00±266.92	**2.52±1.17	***27.50±12.66	**38.00±9.04	4.91±1.34	205.86±55.01
Tachycardia	445.00±0.00	/	/	/	7.88±0.00	227.20±0.00
**Symptomatic therapy**						
No	/	/	/	/	/	/
Yes	614.88±1245.54	1.96±2.23	15.51±8.29	29.21±5.19	5.71±1.58	226.10±110.01
**Anticoagulants**						
No	1174.75±2317.35	1.92±1.12	20.87±12.22	33.30±7.82	5.33±1.48	223.97±82.95
Yes	458.44 659.44	1.97±2.48	13.94±6.28	28.15±3.83	5.84±1.60	226.77±117.73
**Corticosteroids**						
No	*693.28±1819.95	1.31±0.46	14.31±5.05	*31.41±5.94	**5.03±1.46	212.29±74.21
Yes	*575.67±841.97	2.49±2.91	16.49±10.29	*26.86±2.94	**6.15±1.51	233.19±124.34

**Chloroquine**						
No	1174.75±2317.35	1.92±1.12	20.87±12.22	33.30±7.82	5.33±1.48	223.97±82.95
Yes	458.44±659.44	1.97±2.48	13.94±6.28	28.15±3.83	5.84±1.60	226.77±117.73
**Favipiravir**						
No	746.65±1539.94	***1.90±2.50**	14.29±7.79	29.11±3.66	5.59±1.68	226.82±90.49
Yes	388.38±298.65	***2.04±1.94**	16.98±8.93	29.35±6.79	5.87±1.44	225.14±132.67
**Ceftriaxone**						
No	807.17±858.01	1.15±0.15	12.38±1.25	27.77±3.71	5.02±0.71	234.54±143.00
Yes	600.63±1272.29	2.08±2.37	15.97±8.80	29.38±5.37	5.75±1.61	225.36±107.51
**Levofloxacin**						
No	684.97±1411.78	2.09±2.53	14.95±7.33	29.72±5.51	5.65±1.69	226.52±112.11
Yes	394.58±354.99	1.58±1.01	17.10±11.05	27.63±3.96	5.94±0.99	224.85±105.41
**Vancomycin**						
No	524.40±762.71	1.93±2.31	***14.56±6.68**	29.43±5.32	5.62±1.63	221.05±96.76
Yes	1240.00±2913.19	2.22±1.60	***24.30±17.36**	26.30±0.57	6.30±1.05	261.44±178.68
**Meropenem**						
No	467.60±557.95	2.02±2.43	14.74±7.06	***29.89±5.45**	5.61±1.72	215.90±84.67
Yes	1179.44±2485.42	1.71±1.16	18.70±12.58	***26.00±1.51**	6.01±0.99	261.58±169.08
**Metronidazole**						
No	651.20±1362.47	2.24±2.55	16.44±9.49	29.72±5.51	5.61±1.65	215.65±92.29
Yes	465.29±551.00	1.17±0.06	12.83±0.64	27.90±4.30	6.08±1.24	264.41±155.67
**Tocilizumab**						
No	641.59±1295.15	2.01±2.30	15.65±8.56	29.39±5.35	5.67±1.61	224.76±108.37
Yes	309.57±178.98	1.22±0.07	13.40±0.71	26.95±0.78	6.29±0.96	246.16±140.69

*INR - International Normalized Ratio; aPTT - Activated Partial Thromboplastin Time; ECG – Electrocardiogram r - correlation coefficient; p - statistical significance; bold values indicate statistical significance; *p<0.05; **p<0.01; ***p<0.001 (Chi-square, Mann-Whitney U test)*

Supplementary Table S2. Correlation of laboratory parameters with coagulation status in patients with COVID-19 infection hospitalized at the Kosovska Mitrovica Health Center.

Laboratory parameters (reference values)		D-dimer (<500 ng/mL)	INR	Prothrombin time (s)	aPTT (s)	Fibrinogen (2.0–5.0 g/L)	Platelets (150–450 × 10^9/L)
C-reactive protein (<5 mg/L)	r	**0.219***	-0.098	0.036	-0.335	**0.626****	0.070
	p	**0.042**	0.600	0.849	0.076	**0.001**	0.473
Sedimentation (mm/h)	r	**0.413****	0.033	0.058	-0.198	**0.501****	**0.295****
	p	**0.001**	0.859	0.757	0.311	**0.001**	**0.003**
Leukocytes (3.71–10.67 × 10^9/L)	r	**0.296****	0.065	0.133	-0.192	0.124	**0.433****
	p	**0.006**	0.730	0.474	0.319	0.312	**0.001**
Lymphocytes (18.94–46.71%)	r	-0.162	-0.025	-0.094	**0.493****	**-0.593****	-0.109
	p	0.144	0.897	0.620	**0.008**	**0.001**	0.263
Monocytes (2.00–12.00%)	r	-0.118	-0.208	-0.269	**0.438***	**-0.301***	-0.022
	p	0.287	0.271	0.151	**0.020**	**0.013**	0.824
Neutrofili (40.62–71.65%)	r	0.163	0.164	0.211	**-0.526****	**0.546****	0.099
	p	0.141	0.388	0.263	**0.004**	**0.001**	0.311
Eosinophils (0.74–7.00%)	r	0.054	-0.077	-0.053	0.362	**-0.469****	0.125
	p	0.634	0.710	0.797	0.082	**0.001**	0.226
Basophils (0.01-2.00%)	r	0.155	-0.335	**-0.451***	0.002	0.019	**0.450****
	p	0.170	0.094	**0.021**	0.994	0.889	**0.001**
Erythrocytes (3.87–5.68 × 10^{12}/L)	r	-0.071	-0.205	-0.306	-0.219	-0.046	-0.077
	p	0.514	0.268	0.094	0.254	0.706	0.421
Hemoglobin (120–175g/L)	r	-0.092	-0.141	-0.296	-0.180	-0.039	-0.165
	p	0.401	0.449	0.106	0.350	0.750	0.082
Hematocrit (0.35–0.50 L /L)	r	-0.097	-0.186	-0.347	-0.235	-0.065	-0.122
	p	0.383	0.324	0.060	0.228	0.603	0.209
Feritin (30–400 μg/L)	r	-0.124	0.171	-0.021	-0.202	0.182	0.104
	p	0.477	0.527	0.940	0.488	0.363	0.519
Albumins (41–51 g/L)	r	**-0.320****	-0.053	-0.233	0.258	**-0.293***	-0.065
	p	**0.003**	0.778	0.207	0.177	**0.015**	0.501
Proteins (61–88 g/L)	r	-0.081	-0.134	-0.257	-0.080	0.043	-0.056
	p	0.469	0.480	0.170	0.685	0.730	0.576
Alkaline phosphatase (30–120 U/L)	r	0.217	-0.318	-0.270	-0.015	0.055	**0.216***
	p	0.055	0.087	0.149	0.937	0.671	**0.031**
Amilase (23–91 U/L)	r	0.129	-0.115	-0.111	-0.096	0.132	0.013
	p	0.497	0.609	0.623	0.671	0.530	0.939

Direct bilirubin (<3.4 μmol/L)	r	0.127	-0.014	0.046	-0.282	**0.330****	-0.092
	p	0.251	0.940	0.811	0.138	**0.007**	0.352
Total bilirubin (<21 μmol/L)	r	0.131	-0.007	0.183	-0.286	0.138	-0.164
	p	0.242	0.971	0.334	0.132	0.274	0.098
Cholesterol (<5.2 mmol/L)	r	0.041	**-0.431***	**-0.386***	0.159	-0.200	0.099
	p	0.717	**0.015**	**0.032**	0.410	0.105	0.315
LDL (<3.4 mmol/L)	r	0.357	-0.353	-0.406	0.300	0.406	-0.405
	p	0.432	0.492	0.425	0.624	0.425	0.320
HDL (≥1.55 mmol/L)	r	-0.010	-0.187	-0.244	0.374	-0.179	-0.073
	p	0.964	0.429	0.300	0.114	0.438	0.728
Triglycerides (<1.70 mmol/L)	r	0.100	-0.174	-0.139	0.049	-0.241	0.014
	p	0.372	0.351	0.456	0.802	0.052	0.884
AST (35-50 U/L)	r	**0.232***	0.000	-0.086	-0.168	0.193	0.043
	p	**0.032**	10.000	0.644	0.383	0.113	0.650
ALT (35-50 U/L)	r	0.055	-0.049	-0.149	-0.259	0.079	0.141
	p	0.617	0.794	0.423	0.175	0.521	0.141
GGT (38-55 U/L)	r	0.248	0.074	-0.056	-0.358	0.270	0.180
	p	0.096	0.721	0.788	0.066	0.083	0.168
CK (30-300 U/L)	r	-0.068	0.226	0.304	-0.119	-0.030	-0.078
	p	0.545	0.222	0.096	0.540	0.809	0.432
Urea (2.8–7.2 mmol/L)	r	**0.234***	0.133	0.179	0.044	0.054	0.064
	p	**0.031**	0.492	0.353	0.822	0.660	0.511
Creatinine (58–110 mmol/L)	r	0.118	0.351	0.349	0.175	0.128	0.015
	p	0.281	0.062	0.063	0.374	0.300	0.880
Blood glucose (4.1–6.1 mmol/L)	r	0.107	0.256	0.231	0.135	0.223	-0.071
	p	0.338	0.171	0.220	0.492	0.070	0.474
K^+ (4.5–5.4 mmol/L)	r	0.175	-0.125	-0.141	-0.182	**0.263***	0.049
	p	0.105	0.504	0.449	0.346	**0.029**	0.606
Na^+ (135–147 mmol/L)	r	0.056	-0.259	-0.341	0.028	**-0.307***	0.028
	p	0.606	0.160	0.061	0.887	**0.010**	0.771
Ca^{2+} (2.25–2.75 mmol/L)	r	0.126	-0.363	-0.362	0.035	-0.190	0.008
	p	0.416	0.075	0.075	0.869	0.246	0.950
Fe (12–25 μmol/L)	r	-0.207	0.208	0.101	-0.043	**-0.457****	0.180
	p	0.153	0.341	0.647	0.842	**0.002**	0.166

*INR - International Normalized Ratio; aPTT - Activated partial thromboplastin time; LDL - low-density lipoprotein; HDL - high-density lipoprotein; AST - Aspartate aminotransferase; ALT - alanine transaminase; GGT - Gamma-glutamyl transpeptidase; CK - Creatine kinase; K^*- Potassium; Na^+- Sodium; Ca^{++}- Calcium; Fe – iron; p –statistical significance; bold values indicate statistical significance; *p<0.05; **p<0.01; ***p<0.001; r- correlation coefficient*

Supplementary Table S3. Correlation of cytokine concentrations with coagulation status in patients with COVID-19 infection hospitalized in the Kosovska Mitrovica Health Center.

Cytokine concentrations (pg/mL)		D-dimer (<500 ng/mL)	INR	Prothrombin time (s)	aPTT (s)	Fibrinogen (2.0-5.0 g/L)	Platelets (150-450 × 10^9/L)
IL-2	r	0.128	0.082	0.262	-0.171	0.089	0.001
	p	0.237	0.663	0.155	0.375	0.467	0.992
IL-4	r	0.004	0.070	0.075	-0.256	0.073	-0.065
	p	0.971	0.709	0.689	0.180	0.553	0.497
IL-5	r	**-0.277****	-0.171	-0.228	0.137	0.055	-0.079
	p	**0.010**	0.359	0.218	0.479	0.656	0.410
IL-6	r	**0.223***	0.067	0.189	-0.320	0.146	0.080
	p	**0.038**	0.720	0.309	0.090	0.231	0.407
IL-9	r	-0.051	-0.068	-0.093	0.008	0.118	-0.066
	p	0.639	0.715	0.619	0.968	0.334	0.493
IL-10	r	-0.085	-0.176	-0.163	0.198	-0.043	0.121
	p	0.435	0.344	0.380	0.304	0.723	0.204
IL-13	r	0.035	0.049	0.060	0.008	0.223	-0.009
	p	0.750	0.795	0.750	0.967	0.066	0.925
IL-17A	r	0.125	-0.029	-0.060	-0.252	0.181	0.014
	p	0.248	0.878	0.749	0.187	0.136	0.883
IL-17F	r	0.140	-0.114	-0.222	-0.358	**0.258***	**0.189***
	p	0.194	0.542	0.229	0.056	**0.033**	**0.047**
IL-21	r	-0.034	0.225	0.124	0.091	0.069	-0.036
	p	0.753	0.224	0.507	0.640	0.575	0.704
IL-22	r	0.029	-0.067	0.002	-0.221	0.213	0.135
	p	0.792	0.719	0.992	0.249	0.079	0.157
IFN-γ	r	-0.062	-0.288	-0.326	**-0.537****	0.125	0.041
	p	0.567	0.116	0.074	**0.003**	0.308	0.669
TNF-α	r	0.125	0.012	0.112	-0.008	0.154	-0.054
	p	0.250	0.950	0.547	0.966	0.207	0.573

*IL – interleukin; IFN-γ – interferon gamma; TNF-α – tumor necrosis factor alpha; INR - International Normalized Ratio; aPTT - Activated partial thromboplastin time; r- correlation coefficient; p – statistical significance; bold values indicate statistical significance; *p<0.05; **p<0.01; ***p<0.001*
